# Supplementary material for: Multi-step processing of replication stress-derived nascent strand DNA gaps by MRE11 and EXO1 nucleases
Source: Nat Commun. 2023 Oct 7;14:6265. doi: 10.1038/s41467-023-42011-0 (PMC10560291; doi:10.1038/s41467-023-42011-0)
Supplement: Supplementary file 1 — Supplementary Information [file 41467_2023_42011_MOESM1_ESM.pdf]

# Supplementary Information

## Multi-step processing of replication stress-derived nascent strand DNA gaps by MRE11 and EXO1 nucleases

Hale, Dhoonmoon et al.

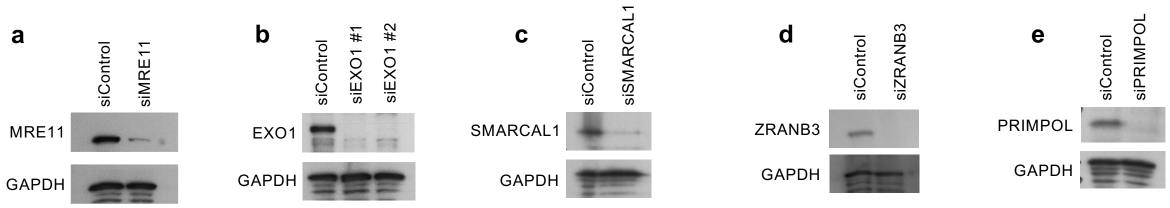


**Supplementary Fig. S1. Confirmation of siRNA-mediated gene knockdown.**

Western blots showing siRNA-mediated knockdown of MRE11 (**a**), EXO1 (**b**), SMARCAL1 (**c**), ZRANB3 (**d**) and PRIMPOL (**e**).

Source data are provided as a Source Data file.

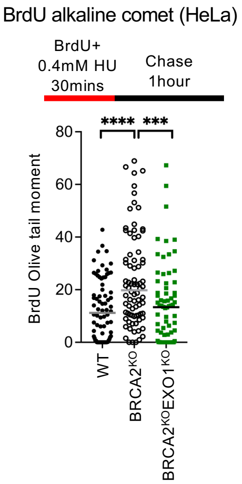


**Supplementary Fig. S2. EXO1 knockout suppresses the accumulation of nascent strand ssDNA gaps in BRCA2-knockout cells.**

BrdU alkaline comet assay showing that EXO1 knockout suppresses the accumulation of replication-associated ssDNA gaps induced by treatment with 0.4mM HU in HeLa-BRCA2$^{KO}$ cells. At least 50 nuclei were quantified for each condition. The median values are marked on the graph. Asterisks indicate statistical significance (Mann-Whitney, two-tailed). A schematic representation of the assay conditions is shown at the top.

Source data are provided as a Source Data file.

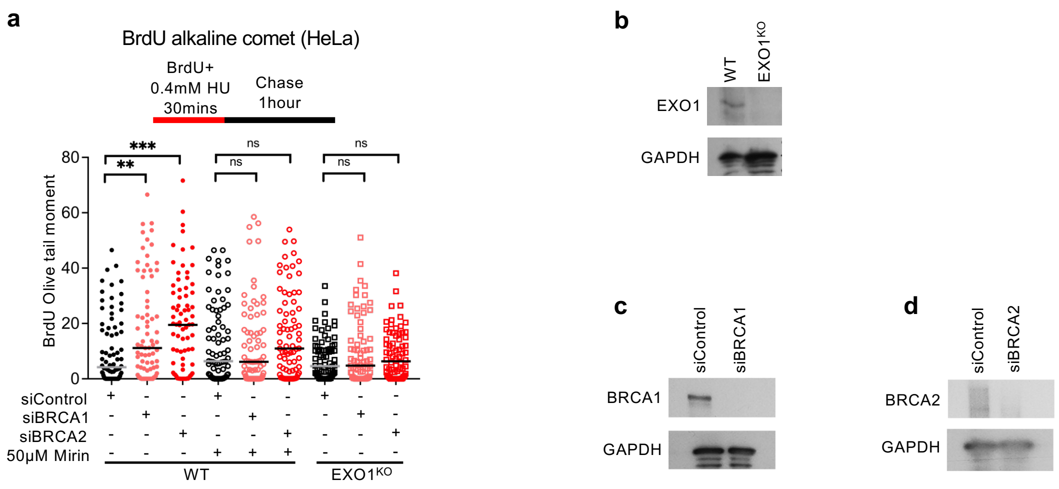


**Supplementary Fig. S3. Impact of EXO1 deletion or MRE11 inhibition on ssDNA gap expansion in BRCA1- and BRCA2-knockdown cells.**

**a.** BrdU alkaline comet assay showing that EXO1 knockout or MRE11 exonuclease inhibition by mirin suppresses the accumulation of replication-associated ssDNA gaps induced by treatment with 0.4mM HU in HeLa cells upon BRCA1- or BRCA2-knockdown. At least 75 nuclei were quantified for each condition. The median values are marked on the graph. Asterisks indicate statistical significance (Mann-Whitney, two-tailed). A schematic representation of the assay conditions is shown at the top.

**b-d**. Western blots showing EXO1 deletion (**b**) and siRNA-mediated knockdown of BRCA1 (**c**) and BRCA2 (**d**).

Source data are provided as a Source Data file.

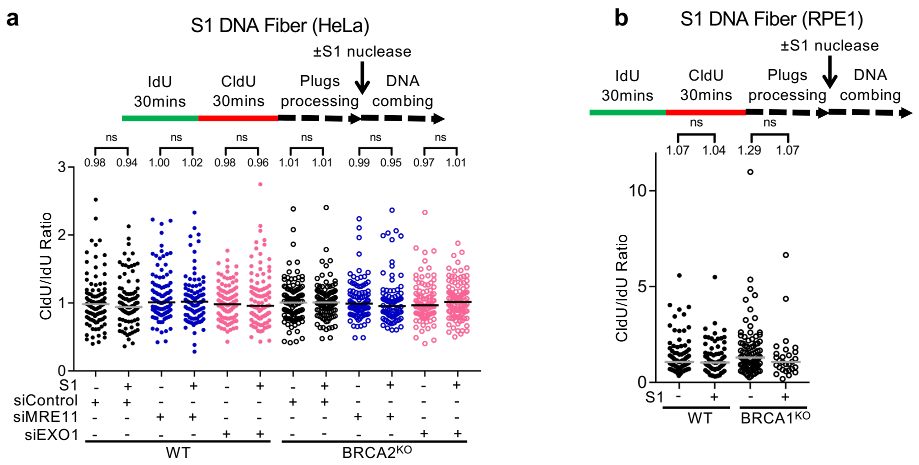


**Supplementary Fig. S4. Loss of BRCA1, BRCA2, EXO1 or MRE11 does not affect CldU/IdU ratios measured by the S1 nuclease assay in the absence of HU treatment**.

The ratios of CldU to IdU tract lengths in HeLa (**a**) and RPE1 (**b**) cells is presented, with the median values marked on the graphs and listed at the top. At least 28 tracts were quantified for each sample. Asterisks indicate statistical significance (Mann-Whitney, two-tailed). Schematic representations of the assay conditions are shown at the top.

Source data are provided as a Source Data file.

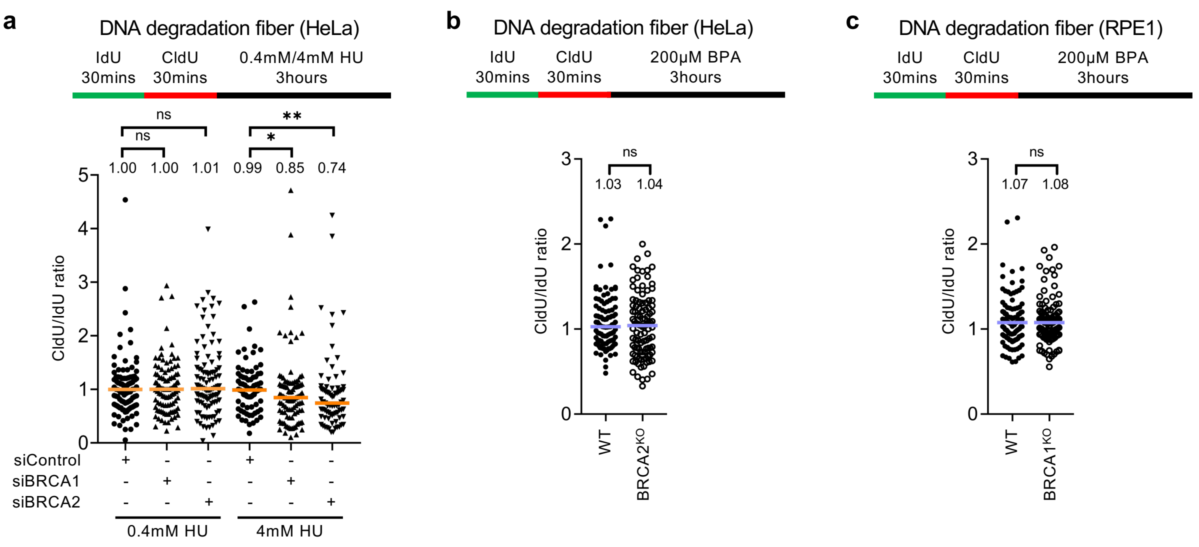


**Supplementary Fig. S5. Treatment of BRCA-deficient cells with 0.4mM HU or 200μM BPA for 3 hours does not induce fork degradation.**

**a.** DNA fiber combing assay showing that treatment with 4mM HU causes fork degradation in HeLa cells upon depletion of BRCA1 or BRCA2, but treatment with 0.4mM HU does not.

**b,c.** DNA fiber combing assay showing that treatment with 200μM BPA does not cause fork degradation in HeLa-BRCA2$^{KO}$ (**b**) or RPE1-BRCA1$^{KO}$ (**c**) cells.

The ratio of CldU to IdU tract lengths is presented, with the median values marked on the graphs and listed at the top. At least 75 (**a**) or 100 (**b,c**) tracts were quantified for each sample. Asterisks indicate statistical significance (Mann-Whitney, two-tailed). Schematic representations of the assay conditions are shown at the top.

Source data are provided as a Source Data file.

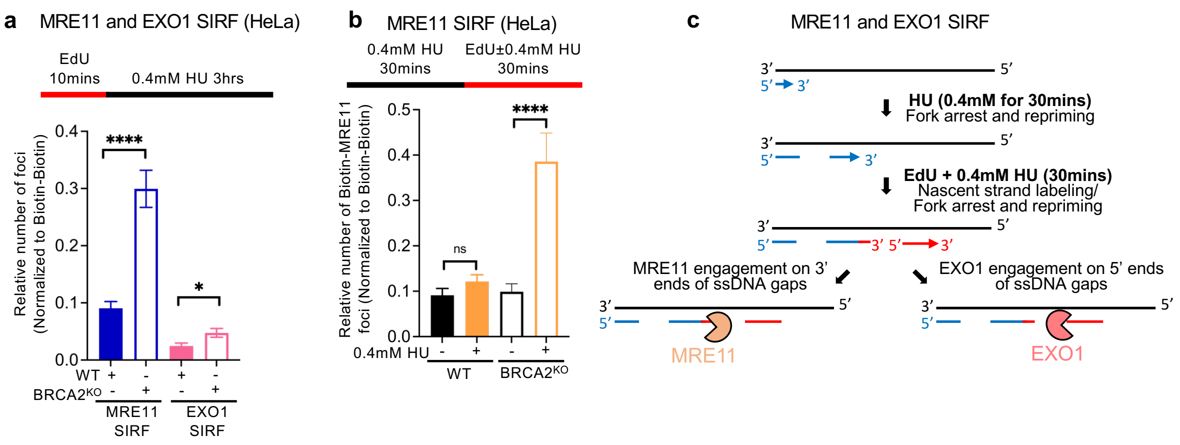


**Supplementary Fig. S6. Detection of MRE11 and EXO1 by SIRF.**

MRE11 (**a**,**b**) and EXO1 (**a**) SIRF experiments using reciprocal labeling schemes, as indicated at the top. EXO1 SIRF signal was low when investigated using the MRE11 labeling scheme (**a**). MRE11 SIRF signal was also detected under EXO1 labeling conditions (**b**), but this is not unexpected since the 3' gap ends may also be EdU-labeled using this scheme (**c**). At least 70 cells were quantified for each condition. Bars indicate the mean values, error bars represent standard errors of the mean, and asterisks indicate statistical significance (t-test, two-tailed, unpaired). Schematic representations of the assay conditions are shown at the top.

Source data are provided as a Source Data file.

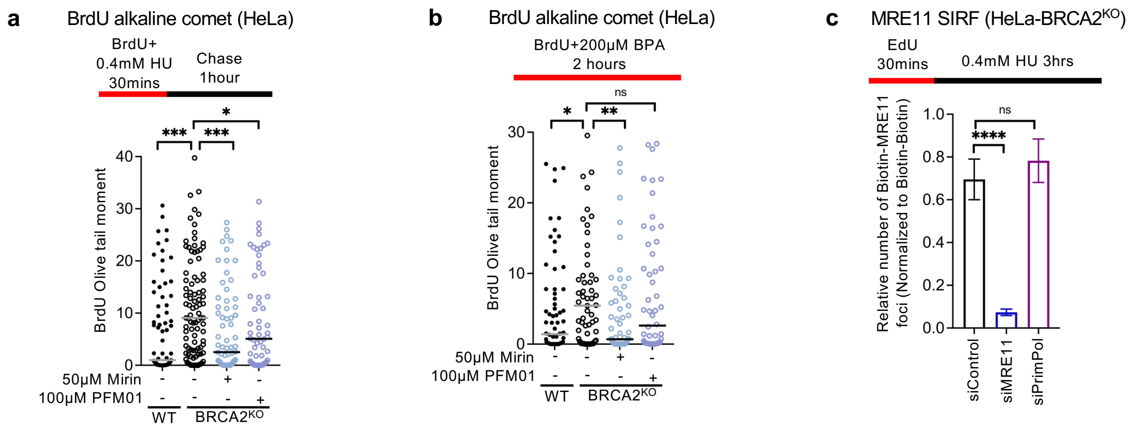


**Supplementary Fig. S7. Processing of ssDNA gaps by MRE11.**

**a,b**. BrdU alkaline comet assay showing that inhibition of MRE11 endonuclease activity using the specific inhibitor PFM01 partially suppressed the ssDNA gaps induced by treatment of HeLa-BRCA2$^{KO}$ cells with HU (**a**) or BPA (**b**), but not to the same extent as the inhibition of its exonuclease activity by mirin. At least 50 nuclei were quantified for each condition. The median values are marked on the graph. Asterisks indicate statistical significance (Mann-Whitney, two-tailed). A schematic representation of the assay conditions is shown at the top.

**c.** SIRF experiment showing that MRE11 binding to nascent DNA upon treatment of HeLa-BRCA2$^{KO}$ cells with 0.4mM HU is not affected by PRIMPOL depletion. At least 95 cells were quantified for each condition. Bars indicate the mean values, error bars represent standard errors of the mean, and asterisks indicate statistical significance (t-test, two-tailed, unpaired). Schematic representations of the assay conditions are shown at the top. Depletion of MRE11 is used as control to confirm the specificity of the SIRF signals observed.

Source data are provided as a Source Data file.

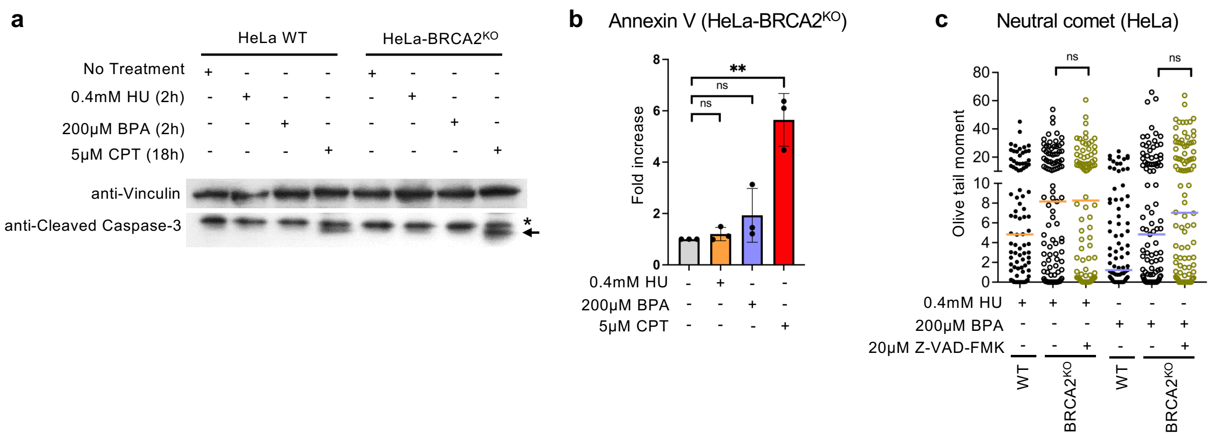


**Supplementary Fig. S8. Treatment with ssDNA gap-inducing agents does not immediately activate apoptosis.**

**a**. Western blots showing that treatment with 0.4mM HU or 200μM BPA for 2 hours does not induce the cleavage of Caspase-3. As a control for apoptosis induction, treatment with 5μM camptothecin (CPT) for 18h was used. The cleaved Caspase-3 band is indicated by the arrow. The asterisk indicated a cross-reactive band.

**b.** Annexin V assays showing that treatment of HeLa-BRCA2$^{KO}$ cells with 0.4mM HU or 200μM BPA for 2 hours does not result in apoptosis induction. The average of three experiments is presented, with standard deviations shown as error bars. Asterisks indicate statistical significance (t-test two-tailed, unpaired).

**c**. Neutral comet assays showing that DSB induced in HeLa-BRCA2$^{KO}$ cells by treatment with 0.4mM HU or 200μM BPA for 2 hours are not suppressed by co-treatment with the apoptosis inhibitor Z-VAD-FMK. At least 84 comets were quantified for each sample. The median values are marked on the graph, and asterisks indicate statistical significance (Mann-Whitney, two-tailed).

Source data are provided as a Source Data file.

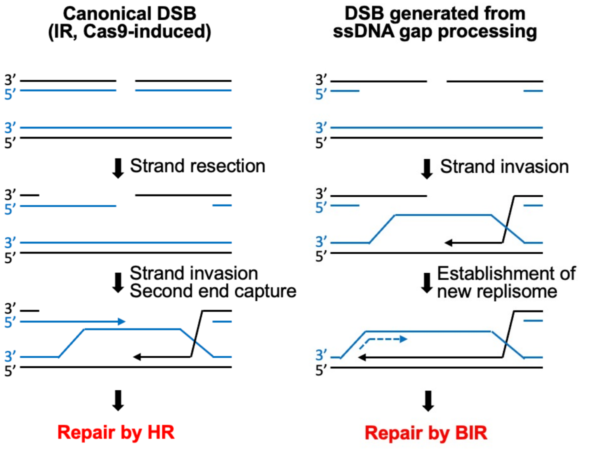


**Supplementary Fig. S9**. **Speculative model for the differential repair of canonical and ssDNA gap-derived double stranded DNA breaks.**

Canonical double strand breaks, such as those induced by ionizing radiation (IR), chemical agents (e.g. bleomycin) or dsDNA-cutting enzymes (e.g. Cas9, SceI) are blunt-ended or have short overhangs. These DSBs can be efficiently repaired through homologous recombination (HR), upon resection of the 5’ ends at the break site to reveal long 3’ overhangs, followed by invasion of the sister chromatid and second end capture. In contrast, DSBs derived from ssDNA gap processing have one long 5’ overhang and one long 3’ overhang, and thus cannot be repaired by HR. Upon sister chromatid invasion by the 3’ end, second end capture cannot take place since the second end does not expose a 3’ overhang. However, continuous extension of the invading strand through DNA synthesis can result in break-induced replication (BIR) upon establishment of a new replisome.
